# Physical Activity and Exercise: Text Mining Analysis

**DOI:** 10.3390/ijerph18189642

**Published:** 2021-09-13

**Authors:** Miquel Pans, Joaquin Madera, Luís-Millan González, Maite Pellicer-Chenoll

**Affiliations:** Department of Physical Education and Sport, FCAFE, Universitat de València, 46010 València, Spain; miquel.pans@uv.es (M.P.); joaquin.madera@uv.es (J.M.); luis.m.gonzalez@uv.es (L.-M.G.)

**Keywords:** physical activity, students, school, university, text mining

## Abstract

It is currently difficult to have a global state of the art vision of certain scientific topics. In the field of physical activity (PA) and exercise, this is due to information overload. The present study aims to provide a solution by analysing a large mass of scientific articles using text mining (TM). The purpose was to analyse what is being investigated in the PA health field on young people from primary, secondary and higher education. Titles and abstracts published in the Web of Science (WOS) database were analysed using TM on 24 November 2020, and after removing duplicates, 85,368 remained. The results show 9960 (unique) words and the most frequently used bi-grams and tri-grams. A co-occurrence network was also generated. ‘Health’ was the first term of importance and the most repeated bi-grams and tri-grams were ‘body_mass’ and ‘body_mass_index’. The analyses of the 20 topics identified focused on health-related terms, the social sphere, sports performance and research processes. It also found that the terms health and exercise have become more important in recent years.

## 1. Introduction

The benefits of physical activity (PA) are well documented, as well as the worldwide effect of the pandemic on physical inactivity [1]. For this, PA for health in young people from primary, secondary and higher education, including both the vulnerable and non-vulnerable populations, is a field of study with enormous relevance. This has reached such a point that an ever-increasing number of articles are published on the subject, even enough to compile special issues as with the present article. The main goal of this paper was thus to mitigate the problem of the present information overload in this field.

If we stop for a moment and observe what is happening in the academic world, we can perceive how science is becoming more and more divided into more specific fields of study. This helps us on many occasions to know a subject in depth, but it also leads to losing a global vision of the state of the art of the topic studied. The number of publications on a specific topic has been growing exponentially since the late 1980s [2]. To date, the ways of knowing the reality and the trends of the importance of what is being studied in a work field involves consulting meta-analysis, systematic reviews or reviews of the topics [3]. However, due to the present large amount of information, the reviews give us only limited information and are usually focused on specific aspects. Obviously, this mass of information has placed research groups in an extreme situation in which it is impossible to read everything that is published. This phenomenon has been called an ‘information overload’ or ‘infobesity’, and occurs when the information supply exceeds the processing capacity [4].

This is a huge problem for many (new) researchers when addressing a new topic, since it is difficult to find the real trends on the matter. In order to respond to this problem, new forms of analysis have been developed to manage such a large volume of information, give a global vision of the topic, and communicate it in a visual and easily understandable manner, without losing its validity and rigor. Text mining (TM) is one of the solutions considered to have a high potential. TM is defined as a set of tools for the processing and extraction of non-trivial knowledge of the amount of text, using different automated techniques. These tools have become significant due to the increased computing capacity the researches have access to and to the storage of large masses of information in digital sources and databases [5]. It is also known as text analytics, intelligent text analysis, text data mining or knowledge-discovery in texts [6]. The goal of TM is to discover unknown information and the results that can be obtained not only generate a single document showing key concepts, it can also help to group, classify and cluster these according to their relationship. TM has also been used recently in fields of knowledge, such as education, business, medicine, biology and biomedicine [7,8]. According to Zhang et al. [9], the TM process generally follows three steps (i.e., pre-processing stage, text mining operations, and post-processing). Text pre-processing is a set of actions that seeks to clean and prepare a natural language text so that it can be analysed through algorithms. Text mining, which is the core part of the process, includes clustering, association rule discovery, analysis of other trends, pattern discovery and knowledge discovery algorithms. Finally, in the post-processing tasks, the knowledge obtained is selected, interpreted and explained.

Previous studies have been conducted on sports science using TM techniques on keywords. These reveal that the trend in this work has moved to the field of rehabilitation and has become a multidisciplinary perspective of the field since the middle of the last century [10]. However, to the best of our knowledge, no work has been conducted to date on a global analysis of the issues addressed in the field of PA related to health and the young and/or vulnerable population addressed in this special issue. In order to obtain valid knowledge from a large mass of textual data, the purpose of this study was twofold: first to examine a large mass of scientific articles for an overall idea of what is happening and being investigated in the field of PA for health in young people at schools and universities, and secondly to discover the dynamics involved and present the topics together with their interrelationships.

To achieve our objectives, the reader can find out in detail how the data was obtained, and it has been explained step by step below. In addition, the steps in the use of the TM technique will be detailed. Next, the results of the analysis will be shown together with the discussion. Finally, some conclusions and future directions are offered.

## 2. Data and Methods

### 2.1. Data Retrieval

This study focuses on the analysis of titles and abstracts published in Web of Science (WOS) database. During October 2020, a group of sport science experts met to establish the theme of this work and outline the structured search that would be carried out later. 

The equation used in the search, taking into account the theme and keywords on the special issue, was the following: (physical_activit* OR exercise) AND (vulnerable_group* OR university* OR school OR student* OR children* OR adolescent* OR youth). The aforementioned search was entered in Web of Science databases using the search fields TITLE, ABSTRACT AND AUTHOR KEYWORDS, refined by type of document (article OR review), on the following databases including in WOS core collection: SCI-EXPANDED, SSCI, A&HCI, CPCI-S, CPCI-SSH, BKCI-S, BKCI-SSH, ESCI, CCR-EXPANDED, IC (for extended descriptions of all of them: https://clarivate.com/webofsciencegroup/solutions/web-of-science-core-collection/ accessed on 7 September 2021). No time or language restrictions were applied. The formal search was carried out on 24 November 2020. For each of the records, the title, abstract, author keywords, year of publication and DOI fields were stored in a CSV file.

### 2.2. Data Pre-Processing

As the first step in the pre-processing, all the duplicated documents were deleted using the DOI (unique identifier) field.

To prepare the text of the documents for further analysis, the standard recommendations used in similar studies were followed [5]. The documents were reduced to tokens (tokenization) and these different actions were carried out in the following order:

- removing all hyperlinks (‘http: // url’);
- punctuation marks and special characters were also removed;
- words were converted to lowercase;
- words that could add noise to the text and did not add content to the documents were eliminated (eg ‘a’, ‘and’, ‘to’), for which the list of stopwords in Matlab’s text analytics toolbox were used;
- because the abstracts of articles occasionally include the copyright and the name of the publisher, these were removed as they do not provide information on the content of the articles;
- words were normalized through a stemming process by which a morphological analysis of the words was carried out to reduce them to their roots using a predefined dictionary; to improve the process part-of-speech details were added indicating whether the words were nouns, verbs, adjectives, etc;
- finally, those words with a character length less than 2 or greater than 20 and those with a frequency in the document corpus of less than 2 were also eliminated.

**Figure 1:**
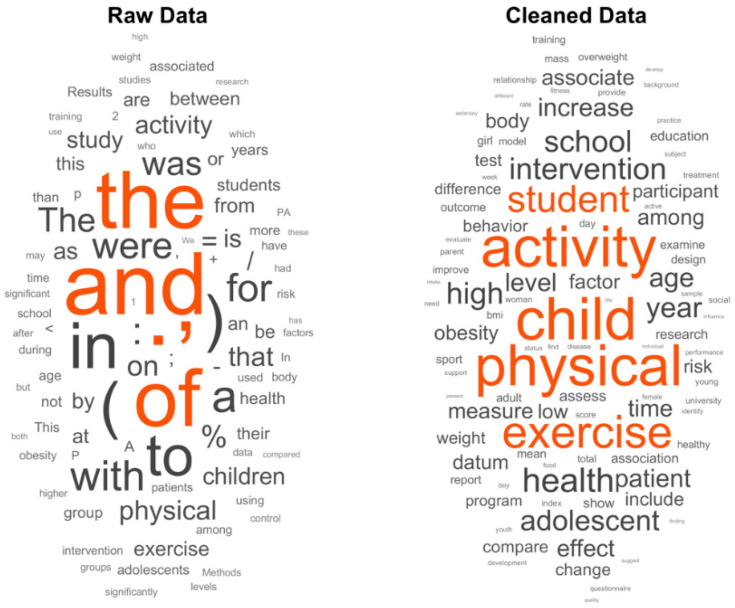
Main words found in the documents before and after pre-processing.

In Figure 1, it can be seen how this pre-processing acts on the corpus of words.

With the resulting tokens, a bag of words (uni-grams) and two bags of grams (bi-grams and tri-grams) were formed. The original documents (raw data) and the associated fields were also stored for later qualitative analysis. 

### 2.3. Descriptive Analysis of Documents and Word Dynamics over the Months

The analysis of the documents began with the description of the recovered documents using only the text and the creation date. A count was made of the number of articles per year. A frequency analysis of the main n-grams was also carried out. An n-gram is a subsequence of n elements of a given sequence of words. The frequency values of the main uni-grams, bi-grams and tri-grams were shown in frequency tables.

On the other hand, to establish the growth and decrease of the most cited words throughout the last 40 years of our research period, the words were represented through a heat map. All term frequency–inverse document frequency (TF-IDF) scores were calculated. TF-IDF measures how important a term is within a document relative to a collection of documents (i.e., corpus of documents). TF-IDF scores a word by multiplying the word’s term frequency (TF) with the inverse document frequency (IDF), where TF is the number of times the term appears in a document compared to the total number of words in the document, and IDF reflects the proportion of documents in the corpus that contains the term.

### 2.4. Co-Ocurrence Analysis and Topics LDA

A co-occurrence analysis was performed with the bi-grams in order to show the number of times that two words appeared simultaneously in a published article. This relationship is established with greater or lesser strength depending on the repetition of this pair of words in a published paper. The co-occurrences of words form a graph in which the nodes are the words and the edges are the co-occurrence relationships between them. As in any graph, the importance of the nodes can be measured through different parameters of centrality [11]. In addition, and to support the analysis of topics that were carried out later, the resulting clusters were calculated through a VOS algorithm. This analysis and the topical analysis (LDA) are complementary.

To identify the topics obtained from the corpus (i.e., collection of summaries) of our data, we applied a latent Dirichlet allocation (LDA) model. The LDA model assumes the existence of a fixed number of latent topics that appear across multiple documents (each of the 85,368 downloaded documents in our case). Each document is characterized by its own mixture of topics, and each topic is characterized by a discrete probability distribution over words; that is, the probability that a specific word is present in a text document depends on the presence of a latent topic.

In our case, the LDA model fulfils a double function; on one hand it extracts the main topics from the corpus studied by different research groups over the years, and on the other it serves as a method of selecting more related documents with certain topics of interest.

To perform the analysis, the ‘filtlda.m’ function implemented in the Matlab text analytics toolbox was used with the bag of previously pre-processed words (uni-gram) to carry out the process. Once the bag of words was ready, the first fundamental step to ensure good cohesion in the resulting topics was to establish the number of topics necessary. To ensure the LDA model’s goodness of fit, a perplexity calculation was previously performed to indicate how well the model described a set of documents. Lower perplexity suggests a better fit. Adjustments were made for 10, 20, 30, and 40 topics. With our data, twenty topics represented the number of topics in the data that yielded the lowest perplexity value, after which an LDA model based on a Gibbs sampling algorithm was implemented.

Of the 20 topics obtained after adjusting the model, those with the highest probabilities of their 10 most representative words were selected. The topic mixtures of each of the documents collected were also calculated to select the most representative documents of each of the selected topics.

We then selected the documents that contained a probability greater than at least 0.4 of belonging to that topic and a qualitative analysis was carried out on these documents to consider them in greater depth.

## 3. Results and Discussion

This is the first study carrying out a global analysis using TM from articles to explore the field of research on PA for health in young people in the different stages of education. All the results obtained from the different analyses are detailed in the following sections.

### 3.1. General Data of the Articles Published

**Figure 2:**
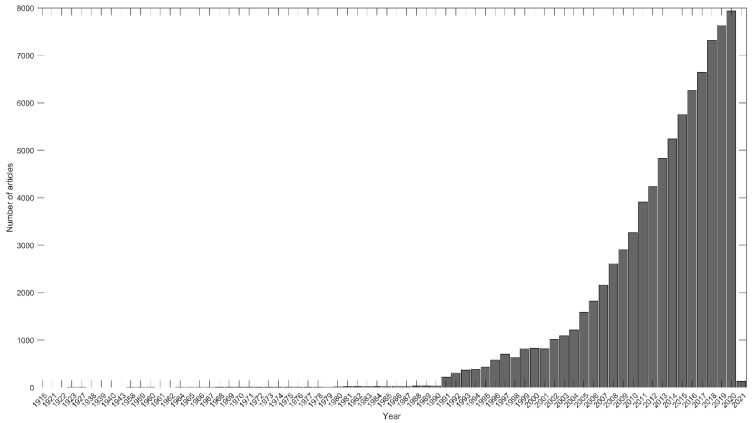
Increase in scientific production in the WOS on the subject of physical exercise in school-age youth and vulnerable populations.

After pre-processing, we analysed a total of 85,368 documents published in the WOS after removing duplicates. Figure 2 shows the exponential increase in the number of articles published since 1990 due to the large number of journals recently included in the WOS in the different categories [2]. Obviously, we cannot dismiss the importance of the field of knowledge we analysed, and it seems that the interest of the scientific community in issues concerning the inclusion of different populations through exercise and sport is also a topical subject [10].

The first article published in our series was by Colby in 1915, entitled “Massage and Remedial Exercises in the Treatment of Children’s Paralyses; Their Differentiation in Use” [12].

### 3.2. Description of the Words Published in the Documents (N-Gram) and Their Dynamics over the Years

In our file, a total of 9960 (unique) words appeared in the total number of documents. Table 1 shows the 20 most repeated uni-grams, bi-grams and tri-grams. Obviously, the words associated with the terms used in the search are those that obtained the highest frequencies (e.g., child, student, etc.). If the words that were entered in the search are not taken into account, we can see that ‘health’ is the first term of importance. Health as a concept seems to be contained in a large number of articles. This vision associating exercise, sport and health is fundamental in the modern conception of physical education [10]. 

The most repeated bi-grams and tri-gram are ‘body_mass’ and ‘body_mass_index’ respectively. It appears that body weight control is an important variable for young populations. This is possibly due to the gradual increase in obesity-related pathologies over the last decade [13,14], such as type II diabetes mellitus [14,15]. This is reinforced by the appearance of two further related terms, ‘blood_pressure’ and ‘overweight_obesity’.

On the other hand, some aspects are linked to the social environment, in which some of the studies analysed. For example, ‘school’, ‘boy_girl’ and ‘high_school’ refer to the different problems generated in educational environments, with special emphasis on aspects linked to gender, which have become important in recent years [16].

**Figure 3:**
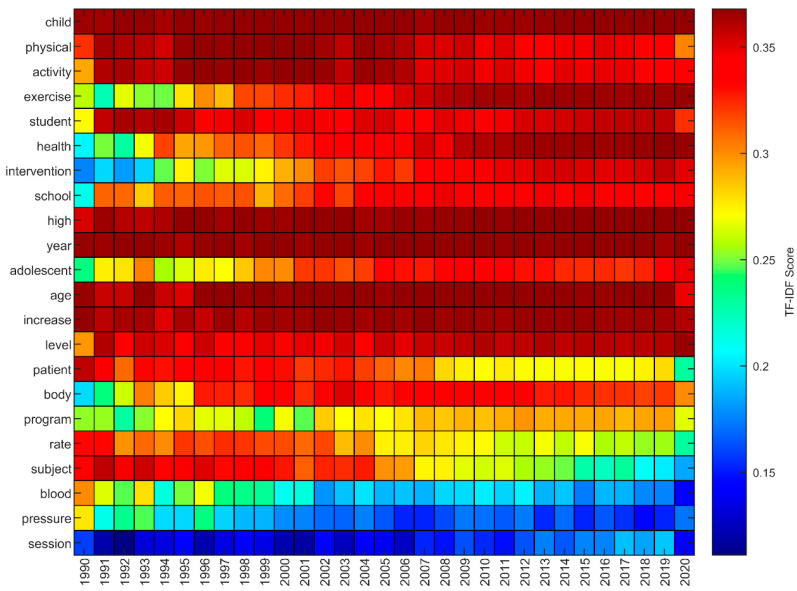
Word dynamics over the last 30 years. TF-IDF represent how important a word is across a set of documents. A high score shows a higher relevance of the word in the document corpus (warmer colors).

However, it is very interesting to analyse how these words have gained or lost importance over the years. Figure 3 shows the dynamics of the uni-grams through their TF-IDF values. This parameter, as already indicated in the material and methods section, gives high scores to the most relevant terms in the documents analysed. Therefore, we can see in the figure that some terms began to lose strength during the first decade of this century. A relevant case is the word ‘patient’. The use of this term is closely linked to the medical conception of physical education and physical exercise that has been a dominant paradigm in the training of professionals working in the field of physical education [10]. Moreover, it seems obvious that the word ‘patient’ may have been replaced by other words, such as ‘participants’, which appears among the most frequently used (i.e., 34,214 times). For those readers who wish to carry out an analysis of less frequently used words, it can be accessed in the Supplementary Materials (see Supplementary Materials Table S1 words).

However, other words have gained weight in the body of knowledge. Two of them must be highlighted for their increase: ‘health’ and ‘exercise’. The former emphasises the importance of health as a concept included in research and the latter refers to a type of physical activity aimed at improving or maintaining physical condition and which is carried out on a regular basis [10].

The latter term shows a contrasting behaviour to ‘physical_activity’. Perhaps we are seeing a change in trend where researchers are no longer only trying to describe the amount of PA that young students engage in, but also how this PA improves their physical fitness [17,18]. 

### 3.3. Word Co-Ocurrences

**Figure 4:**
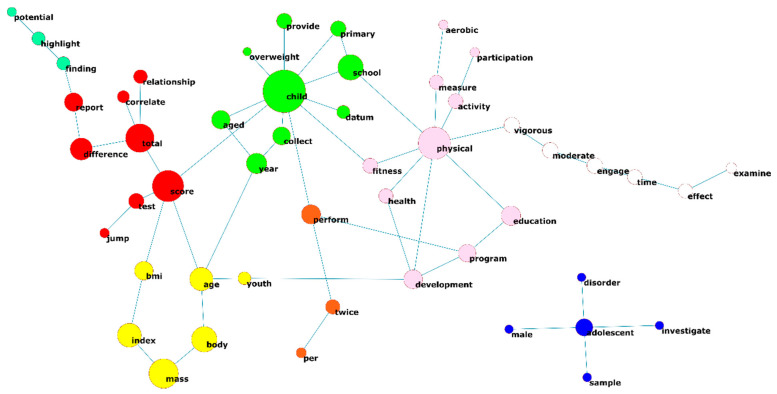
Network of co-occurrences between words (≥4 components). Only those nodes that obtained a minimum of 2000 co-occurrences are represented. The size of the nodes expresses the words’ weighted degree. The different colours of the nodes indicate the cluster membership calculated from a VOS algorithm.

A co-occurrence network was generated with the bi-grams extracted from the articles. This is a non-oriented graph of great complexity and is made up of an infinite number of sub-networks with a highly variable number of components (see Figure 4). As can be seen, one is a network with a high density of words and composed of seven clusters, while another is a sub-network with five components, whose central word is ‘adolescent’.

This co-occurrence analysis allows us to make a first visualization of the possible topics that appear in the LDA analysis. It also reinforces the importance of each of these terms in the frequency analysis. Basically, this analysis based on graph theory is complementary to the LDA analysis and it is therefore discussed at greater length in the following section.

Table 2 shows the centrality data of the main words involved in the network. The word ‘child’ showed the highest centrality parameters. This term was related more than 50,000 times (weighted degree) to 10 other words (degree). Furthermore, the word showed a central position with respect to the rest of the words and evaluates the accessibility of a given node *i* in the whole network, as it had the highest closeness value. It also served as a link between several themes (betweenness). For all these reasons, ‘child’ is the word with the highest prestige due to its properties, being the word that reaches the highest degrees in centrality values. Although we must not forget that this word is among the words used in the search.

### 3.4. Main Topics Found in the LDA Model

The words found were classified into 20 large thematic clusters through an LDA model, ordered from highest to lowest probability in the total corpus of documents. Each topic is composed of a group of words that have an associated probability of appearing together in different documents (see Supplementary Materials Table S2 documents), where it can be seen that some words have a high probability of representing the topic in the different documents.

**Figure 5:**
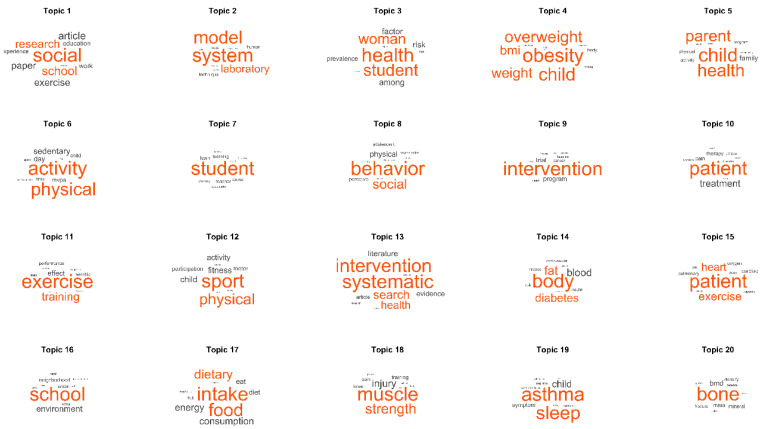
Words most representative of the topics found in the model LDA. Topics are ordered by probability of appearing in the total corpus of documents, topic 1 being the most likely and topic 20 the least likely. The larger word size represents a higher probability of appearing in the documents associated with each topic (for words with a lower probability, see Table S3).

Figure 5 shows the 20 topics whose main word is most likely to appear when talking about the topic they represent. Some of these topics can be easily labelled. For example, topics 5 and 7 show the research related to motor performance and sport while topics 4 and 19 are more focused on treatments applied to patients with various pathologies. Other topics such as 1, 9 and 13 show basic research mainly in the laboratory. However, a qualitative analysis of the main documents selected through our LDA analysis was necessary to characterise the various topics that make up our study.

To better explain the different topics found, we first proceeded to group them into the major areas of interest within the PA and Sport sciences. One of the main approaches, from which half of the topics were derived (topics 3, 4, 6, 10, 12, 14, 15, 17, 19 and 20) refer to the field of Health. Most of the topics classified within this area have as main words conditions or pathologies mostly related to a sedentary lifestyle and physical inactivity. Words such as overweight, obesity, bone density, asthma, diabetes, heart or cardiac occupy the centre of most of these topics. References to terms more related to quality of life or general health can also be found such as dietary, intake, sleep, fitness or health.

This quantity of references to terms related to health is derived from an exponential increase in articles published on recommendations for PA and the use of exercise as a method of preventing pathologies associated with sedentary lifestyles, currently a pandemic in Western societies [1].

Another area in which more topics can be grouped is found within the social sphere (topics 5, 7, 8 and 16), suggesting that physical exercise is highly influenced by the social context [19]. Terms such as school, environment, family and parents refer to the spaces in which the activities of the populations studied take place, which are therefore the socializing agents that can influence their decisions.

A third area reflected in two of the topics found (topics 11 and 18) is more related to sports performance. As we know that PA is closely linked to sports and training, it makes sense to find terms such as training, performance, muscle or strength within these clichés. In performance we also find injury or pain terms, which despite being closely related to training, also touch on the field of health.

Finally, we find some topics (topics 1, 2, 9 and 13) whose central terms can be grouped within the research process. Words such as systematic, research, model, paper, system or laboratory are transversal concepts that could possibly appear in searches in the scientific literature on any subject. In the case of terms such as intervention, program and social, which appear in a central role in these topics, we can deduce that many of the studies in the reviewed literature consist of educational interventions or PA programs in the educational field, such as schools and universities [20].

In addition to the central terms already mentioned, it is worth highlighting the appearance of the term woman in topic 3. This appearance may be due to the increase in research with a gender perspective [21] and we believe that future analysis will possibly include other related terms such as gender or sex.

The absence of terms related to vulnerable populations is striking. We thought that terms such as disability, impairment, special needs would be reflected in the topics. And, it has not been so. This may indicate that in the educational field, the number of papers on a normotype population is much higher than those carried out on students with different abilities. 

## 4. Limitations and Future Research

The main limitation of this study is related to its analysis method. The automatic algorithms used in text mining have a great capacity to analyse large masses of data, but so far cannot fully replace qualitative analysis of texts by experts in the field. It would also be desirable to analyse in languages other than English, even if English is the scientific lingua franca to date. Moreover, the search could have been carried out in other databases with a wider coverage. It should also be noted that the analysis of word dynamics (heat map) may have been influenced by the disproportionate growth in the number of new journals that have appeared in the last 10 years.

Finally, it is important to note that this article highlights the most frequently repeated terms throughout the series analysed, as this strategy makes the major findings clear, leaving aside less frequent terms that may sometimes refer to important new issues in the field.

Beyond these limitations, future works should focus on knowing other possible realities, and may be carried out this analysis in different contexts and languages to observe if there are possible changes in the content due to the languages. Furthermore, it is necessary to delve more deeply into the reasons for the importance of these terms.

## 5. Conclusions

In our work, we have presented a useful aspect on bibliometric analysis. Furthermore, this paper is the first review of the state of the art with respect to exercise and PA related to young and vulnerable populations carried out by text mining analysis.

Since the 1990s, there has been an exponential increase in scientific articles in our area of knowledge. The words associated with the medical conception of physical activity and exercise are the most named. The most cited uni-grams, bi-grams and tri-grams are: ‘child’, ‘body_mass’ and ‘body_mass_index’, respectively; the latter two to the health vision of PA and exercise, and half the topics found refer to health-related terms. If we had not taken into account the words used in the search, the most cited uni-gram would be ‘health’. Therefore, it is observed that the terms that have become most important in recent years are health and exercise. Other issues such as social environment, physical fitness and basic research related topics seem to be of importance in the field, represented by terms such as school, gender, behaviour, performance, training and intervention, system or laboratory.

## Figures and Tables

**Table 1:** Top 20 N-grams found in published papers.

Uni-Gram	Count	Bi-Gram		Count	Tri-Gram			Count
child	134,829	physical	activity	95,660	body	mass	index	10,432
physical	132,159	age	year	14,532	physical	activity	level	5086
activity	131,140	body	mass	12,888	mass	index	bmi	4329
exercise	93,211	risk	factor	11,153	mean	age	year	3740
student	74,059	mass	index	10,898	level	physical	activity	3150
health	65,128	child	adolescent	9808	physical	activity	sedentary	2670
intervention	57,531	physical	education	7481	increase	physical	activity	2646
school	57,325	activity	level	6747	child	aged	year	2227
high	57,036	quality	life	6633	main	outcome	measure	2208
year	55,972	blood	pressure	6622	child	physical	activity	2146
adolescent	55,743	aged	year	6455	physical	activity	among	2103
age	52,267	overweight	obesity	6293	vigorous	physical	activity	2050
increase	48,925	mean	age	5936	bone	mineral	density	1945
level	48,081	boy	girl	5901	physical	activity	child	1920
patient	47,110	heart	rate	5787	physical	activity	intervention	1822
effect	44,719	body	composition	5207	physical	activity	mvpa	1621
time	43,529	overweight	obese	5157	child	age	year	1557
measure	41,476	high	school	5105	diet	physical	activity	1542
among	40,828	sedentary	behavior	4827	high	school	student	1451
associate	39,840	physical	fitness	4822	health-related	quality	life	1439

**Table 2:** Centrality values of the 20 most prestigious words in the co-occurrence network.

Word	Degree	Weighted Degree	Closeness	Betweenness	Proximity Prestige
child	10	50,925	0.268	0.345	0.267
score	5	27,295	0.251	0.302	0.250
school	3	18,194	0.244	0.099	0.243
fitness	2	6195	0.242	0.089	0.241
physical	8	29,026	0.231	0.280	0.230
age	4	15,114	0.225	0.085	0.224
primary	2	6643	0.221	0.000	0.220
perform	3	10,329	0.217	0.077	0.216
development	4	9822	0.216	0.059	0.215
youth	2	4617	0.214	0.046	0.213
aged	2	9579	0.209	0.007	0.208
collect	2	9028	0.209	0.007	0.208
total	4	22,562	0.208	0.182	0.207
program	3	8528	0.204	0.027	0.203
provide	1	6537	0.202	0.000	0.201
datum	1	4848	0.202	0.000	0.201
overweight	1	2006	0.202	0.000	0.201
bmi	2	9671	0.196	0.033	0.196
test	2	6527	0.194	0.029	0.193
year	3	11,696	0.190	0.006	0.189

## Data Availability

All the data are included in Supplementary Materials.

## Supplementary Materials

The following are available online at https://www.mdpi.com/article/10.3390/ijerph18189642/s1, Table S1: words, Table S2: documents, Table S3: Word topic LDA.
